# Technology-Based Mental Health Interventions for Domestic Violence Victims Amid COVID-19

**DOI:** 10.3390/ijerph19074286

**Published:** 2022-04-03

**Authors:** Zhaohui Su, Ali Cheshmehzangi, Dean McDonnell, Hengcai Chen, Junaid Ahmad, Sabina Šegalo, Claudimar Pereira da Veiga

**Affiliations:** 1 School of Public Health, Southeast University, Nanjing 210009, China; 2 Faculty of Science and Engineering, University of Nottingham Ningbo China, Ningbo 315100, China; ali.cheshmehzangi@nottingham.edu.cn (A.C.); hengcai.chen3@nottingham.edu.cn (H.C.); 3 Network for Education and Research on Peace and Sustainability (NERPS), Hiroshima University, Hiroshima 739-8530, Japan; 4 Department of Humanities, Institute of Technology Carlow, R93 V960 Carlow, Ireland; dean.mcdonnell@itcarlow.ie; 5 Prime Institute of Public Health, Peshawar Medical College, Warsak Road, Peshawar 25160, Pakistan; jahmad@piph.prime.edu.pk; 6 Faculty of Health Studies, University of Sarajevo, 71000 Sarajevo, Bosnia and Herzegovina; sabina.segalo@fzs.unsa.ba; 7 School of Management—PPGOLD, Federal University of Parana—UFPR, Curitiba 80210-170, PR, Brazil

**Keywords:** domestic violence, mental health, COVID-19, technology-based interventions, social ecological model

## Abstract

Introduction: Domestic violence is a threat to human dignity and public health. Mounting evidence shows that domestic violence erodes personal and public health, spawning issues such as lifelong mental health challenges. To further compound the situation, COVID-19 and societies’ poor response to the pandemic have not only worsened the domestic violence crisis but also disrupted mental health services for domestic violence victims. While technology-based health solutions can overcome physical constraints posed by the pandemic and offer timely support to address domestic violence victims’ mental health issues, there is a dearth of research in the literature. To bridge the research gap, in this study, we aim to examine technology-based mental health solutions for domestic violence victims amid COVID-19. Methods: A literature review was conducted to examine solutions that domestic violence victims can utilize to safeguard and improve their mental health amid COVID-19. Databases including PubMed, PsycINFO, and Scopus were utilized for the literature search. The search was focused on four themes: domestic violence, mental health, technology-based interventions, and COVID-19. A reverse search of pertinent references was conducted in Google Scholar. The social ecological model was utilized to systematically structure the review findings. Results: The findings show that a wide array of technology-based solutions has been proposed to address mental health challenges faced by domestic violence victims amid COVID-19. However, none of these proposals is based on empirical evidence amid COVID-19. In terms of social and ecological levels of influence, most of the interventions were developed on the individual level, as opposed to the community level or social level, effectively placing the healthcare responsibility on the victims rather than government and health officials. Furthermore, most of the articles failed to address risks associated with utilizing technology-based interventions (e.g., privacy issues) or navigating the online environment (e.g., cyberstalking). Conclusion: Overall, our findings highlight the need for greater research endeavors on the research topic. Although technology-based interventions have great potential in resolving domestic violence victims’ mental health issues, risks associated with these health solutions should be comprehensively acknowledged and addressed.

## 1. Introduction

Domestic violence erodes humanity and global solidarity. Domestic violence or violence against women can be understood as “any act of gender-based violence that results in, or is likely to result in, physical, sexual, or mental harm or suffering to women, including threats of such acts, coercion or arbitrary deprivation of liberty, whether occurring in public or in private life” [1]. It is important to note that domestic violence can happen to both men and women, especially for people who face pronounced disadvantages, such as racial/ethnic or sexual minorities [2,3,4]. That said, to avoid issues such as overgeneration and in light of the high prevalence of domestic violence among women compared to men [3], in this paper, we focused solely on female domestic violence victims. Research conducted by the World Health Organization shows that approximately one in every three women aged 15–49 years is or will become a domestic violence victim [5]. Situations might be considerably worse in low- and middle-income countries [6]. In Colombia, for instance, analyzing data from 2001 to 2009, researchers found that, on average, 54,440 women per year, 149 per day, 6 per hour, or 1 woman every 10 min suffer from sexual violence [7]. These dire statistics could result in grim public health consequences. Mounting evidence shows that domestic violence could exert severe and oftentimes lifelong damage to personal and public health, ranging from anxiety, depression, insomnia, and post-traumatic stress disorders (PTSD) to suicide [8,9,10,11].

COVID-19, along with its resultant crises, has further compounded the situation. A study on 751 women in Tunisia, for instance, shows that reported violence against women rose from 4.4% to 14.8% amid an early COVID-19 lockdown [12]. Worrisome trends have also been confirmed in countries such as the United States (U.S.); data from 36 police and 66 sheriff’s departments across the country show that amid the pandemic, domestic violence cases are on the rise as people retreat to shelters [13]. Analyzing 4618 police reports from the Chicago Police Department, researchers further found that during pandemic lockdowns, domestic violence cases were 64% more likely to happen at residential locations compared to other places [14]. To make situations even more complex, unintended consequences of the COVID-19 pandemic have also been seen in the disruption of healthcare services for domestic violence victims. Ranging from the need for COVID-19 testing and tracing, infections, hospitalizations, and treatments to deaths, the pandemic has forced many nations to allocate most, if not all, resources to address medical emergencies associated with COVID-19, effectively leaving many non-COVID-19-related medical needs, such as mental health services for domestic violence victims, in limbo [15]. By interviewing health departments across the U.S., researchers further found that at least 220 departments had either temporarily or permanently cancelled their non-COVID-related public health services, directly causing a spike in reports of abuses [16].

One way to lessen the impact of COVID-19 and its subsequent medical resource crises on vulnerable populations, such as domestic violence victims, is via finding of alternative health solutions, such as technology-based interventions, that can be accessed virtually without relying on strained healthcare resources. Technology-based interventions can be understood as “the use of technology to design, develop, and/or deliver health promotion contents and strategies that aim to induce or improve positive physical or psychological health outcomes” in victims [17]. Previous evidence shows that compared to other low-scale or low-intensity in-person solutions (e.g., single counseling sessions with a physician), technology-based mental health interventions perform similarly or even better in improving domestic violence victims’ health outcomes [18,19,20,21,22]. For instance, in a randomized controlled trial on interventions with the same content but delivered to the victims differently, i.e., in-person compared to online, findings showed that online-delivered interventions induced greater improvements in domestic violence victims’ mental health [18]. However, although useful insights are available, little is known about the state-of-the-art development of technology-based mental health interventions for domestic violence victims amid the pandemic. Thus, to bridge the research gap, in this study, we aim to identify technology-based mental health solutions for domestic violence victims amid COVID-19.

## 2. Materials and Methods

A literature review was conducted to examine technology-based solutions that domestic violence victims can utilize to safeguard and improve their mental health amid COVID-19. In the context of this study, technology-based solutions are defined as the use of technology to design, develop, and deliver programs and/or strategies that could help improve domestic violence victims’ mental health and wellbeing [23]. In other words, in contrast to in-person interventions, technology-based solutions do not require domestic violence victims’ physical presence to receive and utilize the interventions. Databases including PubMed, PsycINFO, and Scopus were utilized for the literature search. The search was focused on four themes: domestic violence, mental health, technology-based interventions, and COVID-19. An example search query applied to PubMed can be found in Table 1. A reverse search of pertinent references was conducted in Google Scholar. To ensure the review included the most updated insights in the analysis, validated news reports were also examined. The search was confined to articles published in English between 11 March 2020 and 17 October 2021. The inclusion criteria are listed in Table 2. Articles were excluded if they were (1) not published in English, (2) not focused on the COVID-19 pandemic, (3) not conducted in the context of domestic violence, (4) not focused on technology-based interventions, or (5) not offering practical insights into mental health solutions.

### The Social Ecological Model

**Figure 1:**
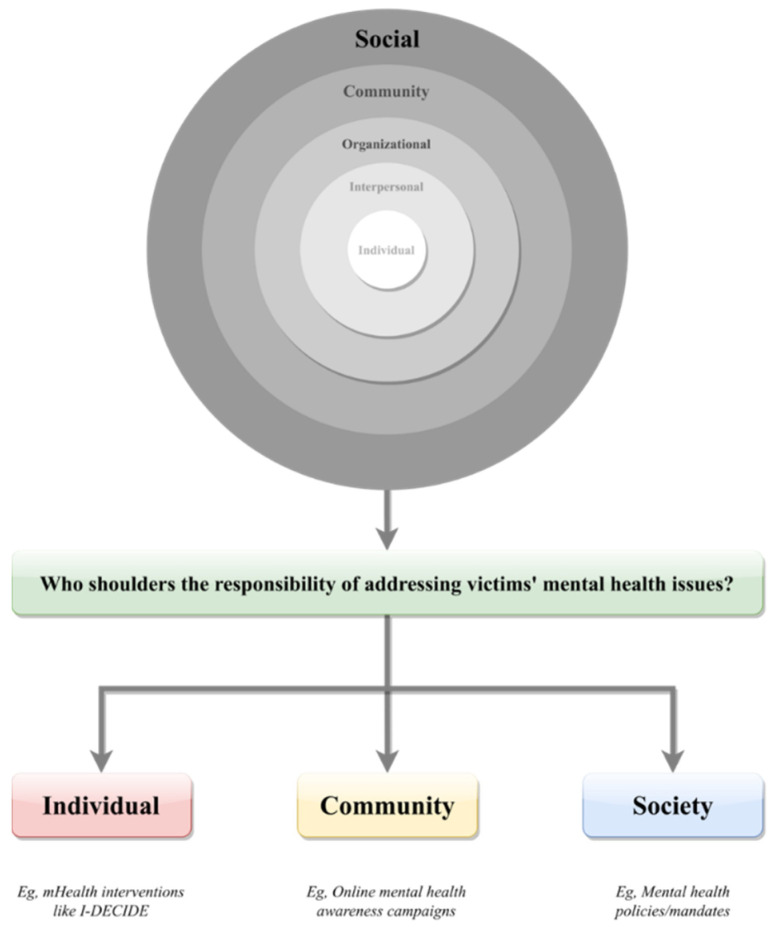
A schematic representation of the modified social ecological model.

The social ecological model was adapted and utilized to theoretically and systematically structure the review findings. The model posits that personal and public behaviors are often impacted by a confluence of social interactions that can be categorized into five levels of influences: intrapersonal, interpersonal, community, organizational, and social [19]. As behavioral sciences evolve, many iterations of the model have been developed [24], largely rooted in the need to address the unique characteristics of individual research contexts (e.g., the influence of the pandemic [25]). In this study, we adapted the original social ecological model to better shed light on our research question. Overall, we tailored the social ecological model’s levels of influences into three levels: individual, community, and society (Figure 1). This categorization was developed in line with the literature, which often places the responsibility of domestic violence as well as the subsequent personal health and safety protection on individual victims or abusers (e.g., women’s ability to leave an abusive relationship [26]), the larger community (e.g., the influence of social support on victims’ wellbeing [27]), or societal influences, such as social norms or policies (e.g., examine domestic violence in light of gender equality [28]).

## 3. Results

A total of nine articles met the selection criteria [29,30,31,32,33,34,35] and were subsequently analyzed to shed light on the research question. Overall, a wide array of technology-based solutions was proposed to address mental health challenges faced by domestic violence victims amid COVID-19, ranging from mHealth self-help tools (e.g., I-DECIDE), online-delivered psychotherapeutic care, and web-based training for healthcare professionals to digital “Doctors Without Borders” services that could provide timely and/or tailored solutions to victims [29,30,31,32,33,34,35]. However, it is important to note that all of these solutions are conceptual proposals, observational evidence, and/or insights from previous non-pandemic-related research, as opposed to programs empirically evaluated amid COVID-19 (e.g., randomized controlled trials). This finding is in line with pre-COVID-19 systematic review findings, which show that there is an imbalance in terms of types of technology-based interventions available to domestic violence victims. For instance, analyzing mobile apps tailored to address domestic violence, researchers found that most of the available apps were targeted one-time solutions (i.e., “emergency” and “avoiding” apps), as opposed to long-term interventions (i.e., “supporting”, “reporting and evidence-building”, and “educating” apps) [36].

When viewing the results via the theoretical lens of the social ecological model, our findings show that most of the interventions were individual-oriented, as opposed to community- or social-level solutions. In other words, although domestic violence is a direct result of abuse and/or assault caused by perpetrators, catalyzed by society’s lack of ability to protect its most vulnerable members, the responsibility of addressing victims’ mental health challenges is largely placed on the victims themselves. Furthermore, most of the articles failed to address the risks associated with utilizing technology-based interventions (e.g., privacy issues) or navigating the online environment (e.g., cyberstalking). In the following section, we will discuss these findings in greater detail.

## 4. Discussion

Domestic violence is a social malaise that jeopardizes humanity and global solidarity. COVID-19 and the ensuing avalanche of crises, ranging from healthcare delivery issues to medical resource shortages, have further exacerbated the challenges faced by domestic violence victims [29]. However, not all hope is lost; compared to in-person solutions, technology-based interventions can be delivered remotely and virtually, effectively bypassing some of the most debilitating barriers introduced by COVID-19 [17,37]. Although COVID-19 has worsened the scale and severity of domestic violence [13,38,39,40], as seen across the pandemic continuum, due to limitations such as physical distancing mandates and lockdown measures, many in-person interventions that were available to domestic violence victims may have been adversely interrupted (e.g., service suspension) [41,42,43]. This, along with the fact that technology-based interventions could offer some victims much-needed anonymity and convenience [29], makes technology-based mental health interventions of particular importance amid the pandemic. In this study, we set out to examine technology-based mental health solutions for domestic violence victims amid COVID-19. Overall, the findings of our study highlight both the benefits and the risks associated with leveraging technological opportunities in addressing domestic violence victims’ mental health challenges amid COVID-19.

### 4.1. Benefits of Technology-Based Interventions

One key finding of our study is that a wide array of technology-based solutions has been proposed by researchers around the world to address mental health challenges faced by domestic violence victims amid COVID-19. Overall, technology-based interventions, compared to non-technology-based interventions, can provide a wider array of advantages to end users, such as greater accessibility (e.g., no need for physical transportation; can be accessed via smartphone, computer, etc.), affordability (e.g., lower cost), availability (e.g., 24/7 access and tailored health solutions), and anonymity (e.g., no need for in-person interactions) [37,44,45,46]. These advantages might be particularly pertinent to domestic violence victims who face pronounced mental health issues, as both health threats—domestic violence and mental health—might be challenging to communicate in person [47].

Traditionally, domestic violence victims can only report their abuse in person or via hotlines, whereas with the help of technology-based interventions, women can capitalize on a wide range of digital tools and online platforms to seek help. Evidence shows that compared to in-person sessions, teleconferences are preferred by domestic violence victims, largely because they “provided a level of control and distance” [48]. This, in turn, can give victims greater freedom in choosing the mental health solutions that are in line with their needs and preferences. Perhaps most importantly, these technology-based interventions give some people access to mental health support who otherwise would not have the opportunity. For instance, many international health and non-profit organizations offer domestic violence victims up-to-date mental health solutions in multiple languages to help such vulnerable communities better cope with pandemic-related stress and beyond (e.g., [49]). This means that when needed, victims have a wide range of access to intervention materials to select from at their own discretion. When high-speed Internet is easily accessible, victims can also utilize tools including smartphone applications (apps), such as I-DECIDE, along with other apps (e.g., WhatsApp or WeChat) and health technologies (e.g., TikTok) to further care for their mental health. However, it is important to note that although seeking information and help via technology-based interventions holds great promise, Herculean tasks are also present for health officials and technologists to ensure that optimal benefits can be delivered via these health solutions. There is also reasonable doubt in terms of whether technology-based solutions can replace more intensive and interactive in-person solutions. This concern is particularly salient in light of the possibility that some people may suffer from mental issues that prevent them from using technology-based solutions, owing to reasons such as paranoiac tendencies and/or fear of technology (e.g., cyber paranoia) [50,51,52].

### 4.2. Risks Associated with Technology-Based Interventions

It is important to highlight that one of the key findings of the current study is that most interventions place the help-seeking responsibility on the victims (i.e., individual level) rather than the larger community (i.e., community level) or societal changes (i.e., social level). In other words, even though domestic violence victims may face a number of challenges above and beyond their mental health issues, they are often expected to initiate the help-seeking actions, have the knowhow to safely navigate the ever-changing technological environment, and possess the capabilities to protect themselves from potential intended and unintended harms that might occur during the process. Essentially, these expectations put the responsibilities of technologists, health officials, and government personnel squarely and simultaneously on the shoulder of the victims, potentially further worsening domestic violence victims’ mental health status, as many technological issues (e.g., cybercrimes) could be extremely difficult to tackle [53,54,55]. Furthermore, as technology advances, ranging from artificial intelligence to sixth-generation technologies (e.g., [56]), it might become increasingly difficult for domestic violence victims with poor eHealth literacy to fully understand and appreciate the content and consequences of technology-based interventions [57,58].

It is important to note that although the cybersphere could be an inclusive and interactive environment for mental health intervention development and distribution, it is often haunted by issues ranging from high dependence on basic infrastructure (e.g., broadband access) and cybercrimes (e.g., cyberbullying and cyberstalking) to social media addiction [59,60,61,62,63]. Studies found that privacy and security issues inherent to technological devices and platforms could influence victims’ appreciation and utilization of technology-based mental health interventions [62], not to mention that in cybersphere, damage related to privacy and data-handling breaches could be extremely difficult to estimate and/or contain. Analyzing 36 top-ranked mobile apps for depression and smoking cessation, researchers found that although approximately 80% of them (29 apps) transmitted user data to companies such as Facebook or Google, only around 41% of them (12 apps) disclosed this practice [64]. Considering the labyrinth of services and devices these two companies hold, it is possible that neither the researchers nor the users will truly comprehend how the data are utilized and by how many sectors.

Recurring evidence also shows that digital addiction, such as social media addiction, could also pose harm to individuals’ mental and physical health, especially vulnerable people, including young adults [65]. Similar to the issue of too much usage, there is also the problem of not having enough access. Partially influenced by poor availability of systematic support, high-speed Internet, and technological devices, research shows that there is a pronounced lack of representation of marginalized and underserved populations (e.g., racial minorities) in mental health technology services [66]. Although digital health solutions are becoming ever-increasingly available, policies that could safeguard their quality to protect the public’s rights and safety are either lax or lagging [67]. Furthermore, legislative hurdles may also hinder victims’ access to quality mental health services via technology-based means. As of October 2021, only twenty-three states in the U.S. have effectively signed on to the Psychology Interjurisdictional Compact (PSYPACT), an agreement that allows cross-state telepsychology services among licensed psychologists [68]—although promising, a far cry from the possibility of having a digital “Doctors Without Borders” system for domestic violence victims [29].

These combined insights suggest that greater governmental support and oversight is needed to ensure the healthy development of technology-based interventions for domestic violence victims. Considering that domestic violence victims often live with their abusers [69], advanced technological solutions, such as AI-enabled facial recognition, can be integrated into various interventions to ensure the content can only be accessed by the victims. Researchers could also use AI technologies, such as natural language processing, to analyze electronic health records to potentially identify victims’ susceptibility to mental health issues before such issues become chronic or permanent [70,71]. Furthermore, by studying large corpora of texts, such as Facebook/Weibo posts, natural language processing technologies could also help government and health officials identify trends, patterns, and incidents of violence and/or mental health emergencies in a timely manner [72,73]. Overall, in light of the capricious nature of COVID-19, it is imperative for government and health officials to apply useful lessons derived from our study, such as taking a proactive and practical yet panoramic approach to developing and deploying technology-based mental health solutions to domestic violence victims, weighing both the pros and cons of such interventions prior to distribution so that optimal intended outcomes can be achieved without causing unintended harms.

### 4.3. The Pronounced Need for More Rigorous Technology-Based Interventions

One alarming finding identified in our investigation is the lack of technology-based interventions that aim to deliver long-term psychotherapy services to domestic violence victims who could be suffering from deep-seated mental health issues [36]. What makes this finding particularly problematic centers on the nature of mental health disorders. It has been long established that, different from acute and symptomatic ailments, mental health issues are often chronic in nature and can be difficult to address [9,74,75]. However, as seen in the results of this study, there is a noticeable lack of long-term technology-based mental health solutions that go beyond one-time or short-term interventions—solutions that could help domestic violence victims better cope with their mental health challenges in a meaningful and sustainable manner. In other words, although technologies can be substantially versatile in providing diverse and timely mental health solutions [23], in the context of mental health solutions for domestic violence victims, they are oftentimes unimaginably utilized to create small-scale and/or low-intensity interventions. This is counterintuitive, especially considering the potential of advanced technologies to address people’s mental health issues (e.g., artificial-intelligence-enabled interventions) [76,77,78] and the clusters of in-person psychotherapy programs that have already accumulated in the literature and the real world [22,78]. In light of these findings, we call for greater research input in developing more robust and rigorous (e.g., high-intensity and long-term) technology-based interventions that can benefit domestic violence victims amid the pandemic and in the long run.

### 4.4. Limitations

Although our study bridges important research gaps in the literature, it is not without limitations. First, this is not a systematic review nor a meta-analysis, which means that our research findings could be limited in their generalizability. The final sample included a limited number of articles, which could further strain the implications of the findings. It is also important to note that during our study process, we identified a salient issue related to the risks associated with using technology and, perhaps most importantly, how it may further expose domestic violence victims to additional threats from abusers and beyond. To address this issue, in light of the suggestions the reviewers kindly shared with us, we have decided to conduct a follow-up study to systematically investigate the potential downsides to technology in the context of domestic violence research.

## 5. Conclusions

Domestic violence violates the fundamentals of humanity: safety, security, agency, and, perhaps most importantly, dignity. COVID-19, along with its resultant crises, has worsened the scale, scope, and severity of domestic violence worldwide. To address the delivery and accessibility challenges caused by the pandemic and the subsequent mandates, technology-based interventions, which could overcome the abovementioned obstacles, are gaining in popularity. Overall, the findings of our study highlight a pronounced need for greater research endeavors to solve the issues identified. Although technology-based interventions have substantial potential to resolve domestic violence victims’ mental health issues, risks associated with such health solutions should be comprehensively acknowledged and thoroughly addressed. For instance, future research could investigate how policy-level support (e.g., increased research funding) can further enrich society’s in-depth understanding, timely development, and victim-centered delivery of technology-based mental health interventions for domestic violence victims.

## Figures and Tables

**Table 1:** Example PubMed search terms.

Theme	Search String
Domestic violence	“domestic violence” [MeSH] OR “domestic violence” [TIAB] OR “intimate partner violence” [MeSH] OR “intimate partner violence” [TIAB] OR “domestic violence” OR “intimate partner violence” OR “violence against women” OR “violence against woman”
Mental health	“mental health” [MeSH] OR “mental health” [TIAB] OR “mental wellbeing” OR “mental well-being” OR “psychological health” OR “psychological wellbeing” OR “psychological well-being”
Technology-based interventions	“technology” [MeSH] OR “technology” [TIAB] OR “eHealth” [TIAB] OR “telemedicine” [MeSH] OR “telemedicine” [TIAB] OR “tele-medicine” [MeSH] OR “tele-medicine” [TIAB] OR “telehealth” [TIAB] OR “tele-health” [TIAB] OR “connected health” [TIAB] OR “digital health” [TIAB] OR “mHealth” [TIAB] OR “mobile health” [TIAB]
COVID-19	“COVID-19” [MeSH] OR “COVID-19” [TIAB] OR “SAS-CoV-2” OR “coronavirus disease 2019”

**Table 2:** Selection criteria.

Category	Inclusion Criteria
Language	English
Focus	Provide insights on technology-based mental health interventions for domestic violence victims
Scope	Published within the context of COVID-19

## Data Availability

Data are available upon reasonable request.

## References

[1] United Nations (1993). Declaration on the Elimination of Violence against Women.

[2] World Health Organization (2010). Preventing Intimate Partner and Sexual Violence against Women: Taking Action and Generating Evidence.

[3] Stöckl H., Devries K., Rotstetin A., Abrahams N., Campbell J., Watts C., Moreno C.G. (2013). The global prevalence of intimate partner homicide: A systematic review. Lancet.

[4] Su Z., McDonnell D., Cheshmehzangi A., Ahmad J., Chen H., Šegalo S., Cai Y. (2022). What “family affair”? Domestic violence awareness in China. Front. Public Health.

[5] World Health Organization (2021). Violence against Women. https://www.who.int/news-room/fact-sheets/detail/violence-against-women.

[6] Sardinha L., Maheu-Giroux M., Stöckl H., Metyer S.R., Galrcía-Moreno C. (2022). Global, regional, and national prevalence estimates of physical or sexual, or both, intimate partner violence against women in 2018. Lancet.

[7] Sanchez O.A., Vivas J.N.L., Cardenas D.R., Cano M.d.P.R. (2011). Sexual Violence against Women in the Context of the Colombian armed Conflict 2001–2009.

[8] Oram S., Khalifeh H., Howard L.M. (2017). Violence against women and mental health. Lancet Psychiatry.

[9] Ellsberg M., Watts C., Yoshihama M., Kiss L., Schraiber L.B., Deyessa N., WHO Multi-Country Study (2008). Intimate partner violence and women’s physical and mental health in the WHO multi-country study on women’s health and domestic violence: An observational study. Lancet.

[10] Indu P.V., Remadevi S., Vidhukumar K., Navas P.M.S., Anilkumar T.V., Subha N. (2017). Domestic violence as a risk factor for attempted suicide in married women. J. Interpers. Violence.

[11] Devries K., Watts C., Yoshihama M., Kiss L., Schraiber L.B., Deyessa N., Heise L., Durand J., Mbwambo J., Jansen H. (2011). Violence against women is strongly associated with suicide attempts: Evidence from the WHO multi-country study on women’s health and domestic violence against women. Soc. Sci. Med..

[12] Sediri S., Zgueb Y., Ouanets S., Ouali U., Bourgou S., Jomli R., Nacef F. (2020). Women’s mental health: Acute impact of COVID-19 pandemic on domestic violence. Arch. Women’s Ment. Health.

[13] Hsu L.-C., Henke A. (2021). COVID-19, staying at home, and domestic violence. Rev. Econ. Househ..

[14] McLay M.M. When “shelter-in-place” isn’t shelter that’s safe: A rapid analysis of domestic violence case differences during the COVID-19 pandemic and stay-at-home orders. J. Fam. Violence.

[15] Cancino R.S., Su Z., Mesa R., E Tomlinson G., Walng J. (2020). The impact of COVID-19 on cancer screening: Challenges and opportunities. JMIR Cancer.

[16] Baker M., Ivory D. (2021). Why Public Health Faces a Crisis across the U.S. https://www.nytimes.com/2021/10/18/us/coronavirus-public-health.html.

[17] Constantino R.E., Braxter B., Ren D., Burroughs J.D., Doswell W.M., Wu L., Hwang J.G., Klem M., Joshi J.B.D., Greene W.B. (2015). Comparing online with face-to-face HELPP intervention in women experiencing intimate partner violence. Issues Ment. Health Nurs..

[18] van Gelder N., Ligthart S., Ten Elzen J., Prins J., van Rosmalen-Nooijens K., Oertelt-Prigione S. (2021). “If I’d had something like SAFE at the time, maybe I would’ve left him sooner.”—Essential features of eHealth interventions for women exposed to intimate partner violence: A qualitative study. J. Interpers. Violence.

[19] Koziol-McLain J., Vandal A.C., Wilson D., Nada-Raja S., Dobbs T., McLean C., Sisk R., Eden K.B., E Glass N. (2018). Efficacy of a Web-Based Safety Decision Aid for Women Experiencing Intimate Partner Violence: Randomized Controlled Trial. J. Med Internet Res..

[20] Tarzia L., Cornelio R., Forsdike K., Hegarty K. (2018). Women’s Experiences Receiving Support Online for Intimate Partner Violence: How Does it Compare to Face-to-Face Support from a Health Professional?. Interact. Comput..

[21] Ogbe E., Harmon S., Bergh R.V.D., Degomme O. (2020). A systematic review of intimate partner violence interventions focused on improving social support and/mental health outcomes of survivors. PLoS ONE.

[22] Su Z., Li X., McDonnell D., A Fernandez A., E Flores B., Wang J. (2021). Technology-Based Interventions for Cancer Caregivers: A Concept Analysis. JMIR Cancer.

[23] Golden S.D., Earp J.A.L. (2012). Social ecological approaches to individuals and their contexts: Twenty years of health education & behavior health promotion interventions. Health Educ. Behav..

[24] Cowan E., Khan M.R., Shastry S., Edelman E.J. (2021). Conceptualizing the effects of the COVID-19 pandemic on people with opioid use disorder: An application of the social ecological model. Addict. Sci. Clin. Pr..

[25] Stone R., Campbell J.K., Kinney D., Rothman E.F. (2021). “He would take my shoes and all the baby’s warm winter gear so we couldn’t leave”: Barriers to safety and recovery experienced by a sample of Vermont women with partner violence and opioid use disorder experiences. J. Rural Health.

[26] Sullivan C.M. (2018). Understanding How Domestic Violence Support Services Promote Survivor Well-being: A Conceptual Model. J. Fam. Violence.

[27] Kuskoff E., Parsell C. (2021). Striving for Gender Equality: Representations of Gender in “Progressive” Domestic Violence Policy. Violence Women.

[28] Su Z., McDonnell D., Roth S., Li Q., Šegalo S., Shi F., Wagers S. (2021). Mental health solutions for domestic violence victims amid COVID-19: A review of the literature. Glob. Health.

[29] Mazza M., Marano G., Lai C., Janiri L., Sani G. (2020). Danger in danger: Interpersonal violence during COVID-19 quarantine. Psychiatry Res..

[30] Fegert J.M., Vitiello B., Plener P.L., Clemens V. (2020). Challenges and burden of the Coronavirus 2019 (COVID-19) pandemic for child and adolescent mental health: A narrative review to highlight clinical and research needs in the acute phase and the long return to normality. Child Adolesc. Psychiatry Ment. Health.

[31] Emezue C. (2020). Digital or Digitally Delivered Responses to Domestic and Intimate Partner Violence During COVID-19. JMIR Public Health Surveill.

[32] Bennett E.R., Snyder S., Cusano J., McMahon S., Zijdel M., Camerer K., Howley C. (2021). Supporting survivors of campus dating and sexual violence during COVID-19: A social work perspective. Soc. Work Health Care.

[33] Bradley N.L., DiPasquale A.M., Dillabough K., Schneider P.S. (2020). Health care practitioners’ responsibility to address intimate partner violence related to the COVID-19 pandemic. Can. Med. Assoc. J..

[34] Barbara G., Facchin F., Micci L., Rendiniello M., Giulini P., Cattaneo C., Vercellini P., Kustermann A. (2020). COVID-19, Lockdown, and Intimate Partner Violence: Some Data from an Italian Service and Suggestions for Future Approaches. J. Women’s Health (Larchmt).

[35] Sauerborn E., Eisenhut K., Ganguli-Mitra A., Wild V. (2021). Digitally supported public health interventions through the lens of structural injustice: The case of mobile apps responding to violence against women and girls. Bioethics.

[36] Su Z., McDonnell D., Liang B., Kue J., Li X., Šegalo S., Advani S., Flores B.E., Wang J. (2021). Technology-based health solutions for cancer caregivers to better shoulder the impact of COVID-19: A systematic review protocol. Syst. Rev..

[37] Su Z., Cheshmehzangi A., McDonnell D., Šegalo S., Ahmad J., Bennett B. (2021). Gender inequality and health disparity amid COVID-19. Nurs. Outlook.

[38] Leigh J.K., Peña L.D., Anurudran A., Pai A. (2022). “Are you safe to talk?”: Perspectives of Service Providers on Experiences of Domestic Violence During the COVID-19 Pandemic. J. Fam. Violence.

[39] Flor L.S., Friedman J., Spencer C.N., Cagney J., Arrieta A., E Herbert M., Stein C., Mullany E.C., Hon J., Patwardhan V. (2022). Quantifying the effects of the COVID-19 pandemic on gender equality on health, social, and economic indicators: A comprehensive review of data from March, 2020, to September, 2021. Lancet.

[40] Morton A., Adams C. (2022). Health visiting in England: The impact of the COVID-19 pandemic. Public Health Nurs..

[41] Panovska-Griffiths J., Szilassy E., Johnson M., Dixon S., De Simoni A., Wileman V., Dowrick A., Emsley E., Griffiths C., Barbosa E.C. (2022). Impact of the first national COVID-19 lockdown on referral of women experiencing domestic violence and abuse in England and Wales. BMC Public Health.

[42] Shah S.S., Mufeed S.A. (2022). Urgency and relevance of feminist social work to curb domestic violence amid COVID-19. Int. Soc. Work.

[43] Tarzia L., Iyer D., Thrower E., Hegarty K. (2017). “Technology doesn’t judge you”: Young Australian women’s views on using the internet and smartphones to address intimate partner violence. J. Technol. Hum. Serv..

[44] Anderson E.J., Krause K.C., Krause C.M., Welter A., McClelland D.J., Garcia D.O., Ernst K., Lopez E.C., Koss M.P. (2019). Web-Based and mHealth Interventions for Intimate Partner Violence Victimization Prevention: A Systematic Review. Trauma Violence Abus..

[45] Murta S.G., Parada P.D.O., Meneses S., Medeiros J.V.V., Balbino A., Rodrigues M.C., Miura M.A., Dos Santos T.A.A., De Vries H. (2020). Dating SOS: A systematic and theory-based development of a web-based tailored intervention to prevent dating violence among Brazilian youth. BMC Public Health.

[46] Clayson P.E., Carbine K.A., Baldwin S.A., Larson M.J. (2019). Methodological reporting behavior, sample sizes, and statistical power in studies of event-related potentials: Barriers to reproducibility and replicability. Psychophysiology.

[47] Thomas C.R., Miller G., Hartshorn J.C., Speck N.C., Walker G. (2005). Telepsychiatry Program for Rural Victims of Domestic Violence. Telemed. J. e-Health.

[48] Women’s Aid (2021). The Survivor’s Handbook: Information in Other Languages. https://www.womensaid.org.uk/the-survivors-handbook/different-languages/.

[49] Mason O.J., Stevenson C., Freedman F. (2014). Ever-present threats from information technology: The Cyber-Paranoia and Fear Scale. Front. Psychol..

[50] Kaya A.Y., Ata F., Aker H., Aiken M. (2022). New Media and Digital Paranoia: Extreme Skepticism in Digital Communication. Handbook of Research on Cyberchondria, Health Literacy, and the Role of Media in Society’s Perception of Medical Information.

[51] Zimaitis I., Degutis M., Urbonavicius S. (2020). Social media use and paranoia: Factors that matter in online shopping. Sustainability.

[52] Winfield A. (2019). Ethical standards in robotics and AI. Nat. Electron..

[53] Keskinbora K.H. (2019). Medical ethics considerations on artificial intelligence. J. Clin. Neurosci..

[54] Hunt X., Tomlinson M., Sikander S., Skeen S., Marlow M., Du Toit S., Eisner M. (2020). Artificial Intelligence, Big Data, and mHealth: The Frontiers of the Prevention of Violence Against Children. Front. Artif. Intell..

[55] Su Z., McDonnell D., Bentley B.L., He J., Shi F., Cheshmehzangi A., Ahmad J., Jia P. (2021). Addressing Biodisaster X Threats with Artificial Intelligence and 6G Technologies: Literature Review and Critical Insights. J. Med. Internet Res..

[56] Jacobson N.C., Bentley K.H., Walton A., Wang S.B., Fortgang R.G., Millner A.J., Coombs G., Rodman A.M., Coppersmith D.D.L. (2020). Ethical dilemmas posed by mobile health and machine learning in psychiatry research. Bull. World Health Organ..

[57] Davison T. (2021). Cyberthreats are going mobile and it’s time to take action. Comput. Fraud. Secur..

[58] Parsons C., Molnar A., Dalek J., Knockel J., Kenyon M., Haselton B., Khoo C., Deibert R., Lab C. (2021). The Predator in Your Pocket: A Multidisciplinary Assessment of the Stalkerware Application Industry.

[59] Jattamart A., Kwangsawad A. (2021). What awareness variables are associated with motivation for changing risky behaviors to prevent recurring victims of cyberbullying?. Heliyon.

[60] Lopes Gomes Pinto Ferreira G., Bailey J., Flynn A., Henry N. (2021). Technology as both a facilitator of and response to youth intimate partner violence: Perspectives from advocates in the global-south. The Emerald International Handbook of Technology-Facilitated Violence and Abuse.

[61] Bauerly B.C., Mccord R.F., Hulkower R., Pepin D. (2019). Broadband Access as a Public Health Issue: The Role of Law in Expanding Broadband Access and Connecting Underserved Communities for Better Health Outcomes. J. Law Med. Ethics.

[62] Huckvale K., Torous J., Larsen M.E. (2019). Assessment of thedata sharing and privacy practices of smartphone apps for depression and smoking cessation. JAMA Netw. Open.

[63] Montag C., Elhai J.D. (2020). Discussing digital technology overuse in children and adolescents during the COVID-19 pandemic and beyond: On the importance of considering Affective Neuroscience Theory. Addict. Behav. Rep..

[64] Schueller S.M., Hunter J.F., Figueroa C., Aguilera A. (2019). Use of Digital Mental Health for Marginalized and Underserved Populations. Curr. Treat. Options Psychiatry.

[65] Pappas S. (2021). Providing Care in Innovative Ways. https://www.apa.org/monitor/2020/01/cover-trends-innovative-ways.

[66] (2021). The Psychology Interjurisdictional Compact. Map. https://psypact.site-ym.com/general/custom.asp?page=psypactmap.

[67] Bradbury-Jones C., Isham L. (2020). The pandemic paradox: The consequences of COVID-19 on domestic violence. J. Clin. Nurs..

[68] Vaci N., Liu Q., Kormilitzin A., De Crescenzo F., Kurtulmus A., Harvey J., O’Dell B., Innocent S., Tomlinson A., Cipriani A. (2020). Natural language processing for structuring clinical text data on depression using UK-CRIS. Évid. Based Ment. Health.

[69] Annapragada A.V., Donaruma-Kwoh M.M., Annapragada A.V., Starosolski Z.A. (2021). A natural language processing and deep learning approach to identify child abuse from pediatric electronic medical records. PLoS ONE.

[70] Ni Y., Barzman D., Bachtel A., Griffey M., Osborn A., Sorter M. (2020). Finding warning markers: Leveraging natural language processing and machine learning technologies to detect risk of school violence. Int. J. Med. Inform..

[71] Subramani S., Wang H., Vu H.Q., Li G. (2018). Domestic violence crisis identification from facebook posts based on deep learning. IEEE Access.

[72] Howard L.M., Trevillion K., Agnew-Davies R. (2010). Domestic violence and mental health. Int. Rev. Psychiatry.

[73] Campbell J.C. (2002). Health consequences of intimate partner violence. Lancet.

[74] Graham S., Depp C., Lee E.E., Nebeker C., Tu X., Kim H.-C., Jeste D.V. (2019). Artificial Intelligence for Mental Health and Mental Illnesses: An Overview. Curr. Psychiatry Rep..

[75] Lovejoy C.A., Buch V., Maruthappu M. (2019). Technology and mental health: The role of artificial intelligence. Eur. Psychiatry.

[76] Kolenik T., Gams M. (2021). Persuasive Technology for Mental Health: One Step Closer to (Mental Health Care) Equality?. IEEE Technol. Soc. Mag..

[77] Ragavan M.I., Thomas K., Medzhitova J., Brewer N., Goodman L.A., Bair-Merritt M. (2019). A systematic review of community-based research interventions for domestic violence survivors. Psychol. Violence.

[78] Johnson L., Stylianou A.M. (2020). Coordinated Community Responses to Domestic Violence: A Systematic Review of the Literature. Trauma Violence Abus..

